# Development and Implementation of a Personal Virtual Assistant for Patient Engagement and Communication in Postsurgical Cancer Care: Feasibility Cohort Study

**DOI:** 10.2196/64145

**Published:** 2025-02-18

**Authors:** Miguel Bargas-Ochoa, Alejandro Zulbaran-Rojas, M G Finco, Anthony B Costales, Areli Flores-Camargo, Rasha O Bara, Manuel Pacheco, Tina Phan, Aleena Khichi, Bijan Najafi

**Affiliations:** 1 Digital Health Access Center (DiHAC), Michael E DeBakey Department of Surgery, Baylor College of Medicine, 7200 Cambridge st, Houston, TX, 77030, United States; 2 Department of Physical Therapy, University of North Texas Health Science Center, Fort Worth, TX, United States; 3 Division of Gynecologic Oncology, Dan L Duncan Comprehensive Cancer Center, Baylor College of Medicine, Houston, TX, United States; 4 Center for Advanced Surgical & Interventional Technology (CASIT), Department of Surgery, David Geffen School of Medicine, University of California, Los Angeles, 700 Westwood Plaza, Suite 2200, Los Angeles, CA, 90095, United States, 1 (424) 467-7127

**Keywords:** digital health, personal virtual assistant, remote patient monitoring, surgical oncology, posthospital discharge, postoperative support, medication adherence postsurgery, patient engagement, mHealth, mobile health

## Abstract

**Background:**

Cancer-care complexity heightens communication challenges between health care providers and patients, impacting their treatment adherence. This is especially evident upon hospital discharge in patients undergoing surgical procedures. Digital health tools offer potential solutions to address communication challenges seen in current discharge protocols. We aim to explore the usability and acceptability of an interactive health platform among discharged patients who underwent oncology-related procedures.

**Methods:**

A 4-week exploratory cohort study was conducted. Following hospital discharge, a tablet equipped with an integrated Personal Virtual Assistant (PVA) system was provided to patients who underwent oncology-related procedures. The PVA encompasses automated features that provide personalized care plans, developed through collaboration among clinicians, researchers, and engineers from various disciplines. These plans include guidance on daily specific assignments that were divided into 4 categories: medication intake, exercise**,** symptom surveys, and postprocedural specific tasks. The aim was to explore the acceptability of the PVA by quantification of dropout rate and assessing adherence to each care plan category throughout the study duration. The secondary aim assessed acceptability of the PVA through a technology acceptance model (TAM) questionnaire that examined ease of use, usefulness, attitude toward use, and privacy concerns.

**Results:**

In total, 17 patients were enrolled. However, 1 (5.8%) patient dropped out from the study after 3 days due to health deterioration, leaving 16/17 (94.2%) completing the study (mean age 54.5, SD 12.7, years; n=9, 52% Caucasian; n=14, 82% with a gynecological disease; n=3, 18% with a hepatobiliary disease). At the study end point, adherence to care plan categories were 78% (SD 25%) for medications, 81% (SD 24%) for exercises, 61% (SD 30%) for surveys, and 58% (SD 44%) for specific tasks such as following step-by step wound care instructions, managing drains, administering injectable medications independently, and performing pelvic baths as instructed. There was an 80% patient endorsement (strongly agree or agree) across all TAM categories.

**Conclusion:**

This study suggests the potential acceptability of the PVA among patients discharged after oncology-related procedures, with a dropout rate of less than 6% and fair-to-good adherence to tasks such as medication intake and exercise. However, these findings are preliminary due to the small sample size and highlight the need for further research with larger cohorts to validate and refine the system.

## Introduction

Ineffective communication between cancer patients and health care providers can result in heightened distress, compromised quality of life [1], reduced treatment adherence [2,3], and suboptimal quality of care [4]. The complexity of cancer care, coupled with the necessity for a multidisciplinary approach, exacerbates communication challenges in this population [4,5]. For instance, multiple health care professionals including surgeons, medical and radiation oncologists, pathologists, nurses, physical therapists, social workers, and nutritionists, amongst others, can be involved in one single treatment plan [6]. This multidisciplinary approach, while essential to provide comprehensive care, often results in multiple hospital follow-ups, varied medication regimens and heterogeneous instructions, which can lead to misunderstandings, failure of cancer treatment protocols, and physical and psychological harm [4].

These communication issues in cancer patients become especially evident in those undergoing surgical procedures and transitioning from hospital to home settings [7]. Upon hospital discharge, cancer patients are commonly provided with a list of extensive instructions based on written or printed summaries, which are lengthy and tedious [8-11], making it difficult to understand and follow [12,13]. In recent years, there has been a shift toward using computer generated programs to create quick and interactive materials in the form of educational websites, audio, and videos [14]. However, they still fall short in providing interactive and bidirectional communication with health providers.

During the COVID-19 pandemic, digital health emerged as an alternative to address communication challenges through technology platforms and remote health monitoring [15]. Digital tools such as telemonitoring, telemedicine, mobile health apps, and wearable devices [16] became useful tools for improving treatment compliance, symptom management, and patient communication. However, as these tools become more widely adopted, it has become evident that numerous challenges must be addressed to improve their usability, accessibility, and effectiveness, particularly in cancer care. Key challenges in adopting digital technologies for cancer patients include disparities in technology literacy, poor integration into clinical workflows, time-intensive processes, and limited bidirectional communication. Furthermore, content may be biased if created by those without health-related expertise. To address these challenges, a collaboration between academia (Baylor College of Medicine, Houston) and industry (Smartek21, Seattle) developed an interactive digital platform, called Personal Virtual Assistant (PVA). The PVA is designed to virtually coach patients in adhering to their postoperative care plans and enhance communication after hospital discharge. This exploratory study examined the usability and acceptability of the PVA among patients undergoing oncology-related procedures, aiming to identify its successful components, adoption barriers and areas for improvement.

## Methods

### Personal Virtual Assistant Development

The PVA streamlines posthospital discharge care plans through 3 key elements: an interactive patient platform, a care provider portal, and a secure cloud backend interface.

**Figure 1.**
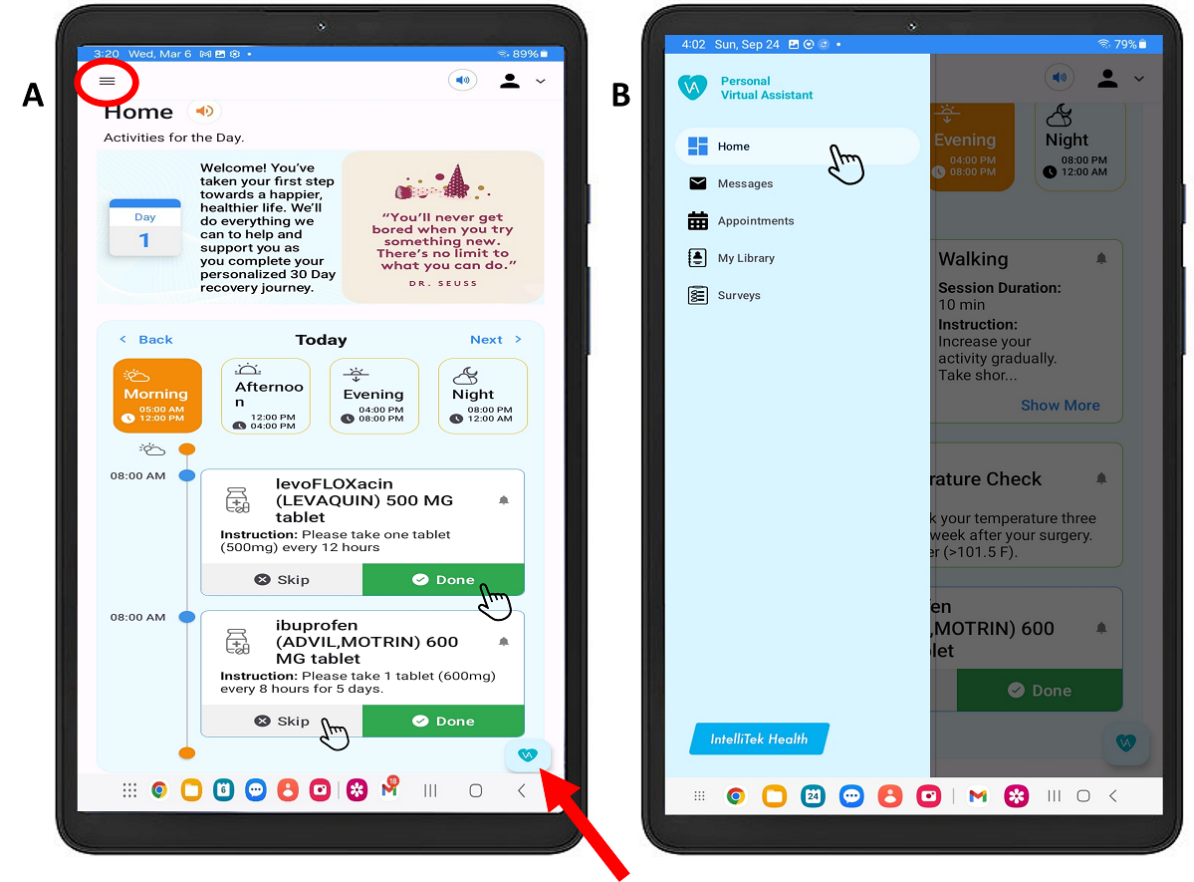
Personal Virtual Assistant displaying the patients’ portal home screen. (**A**) Personal Virtual Assistant displaying the home screen once opened. When scrolling down, all 4 categories (medications, exercises, symptom surveys, and specific tasks) are seen. The red circle represents the button to navigation sidebar and the red arrow depicts the voice-command button. (**B**) Navigation sidebar options displaying additional navigation tools such as messages, appointments, my library, and symptom surveys.

The Interactive Patient Platform uses an app integrated into a tablet that works through internet connection. Upon launching the app, the home screen displays the patient daily care plan (Figure 1A), which can include “prescribed medications,” “exercises,” “symptom surveys,” and “postprocedural specific tasks.” The assignments that appear on the home screen depend on the time and day of recovery. Patients have the option to navigate through these assignments by manual selection or by using natural language voice-commands upon pressing the “voice-command” button and speaking aloud the category name. In addition, the app has an alert notification system to remind patients to complete the assignments by marking each one as “done.”

**Figure 2.**
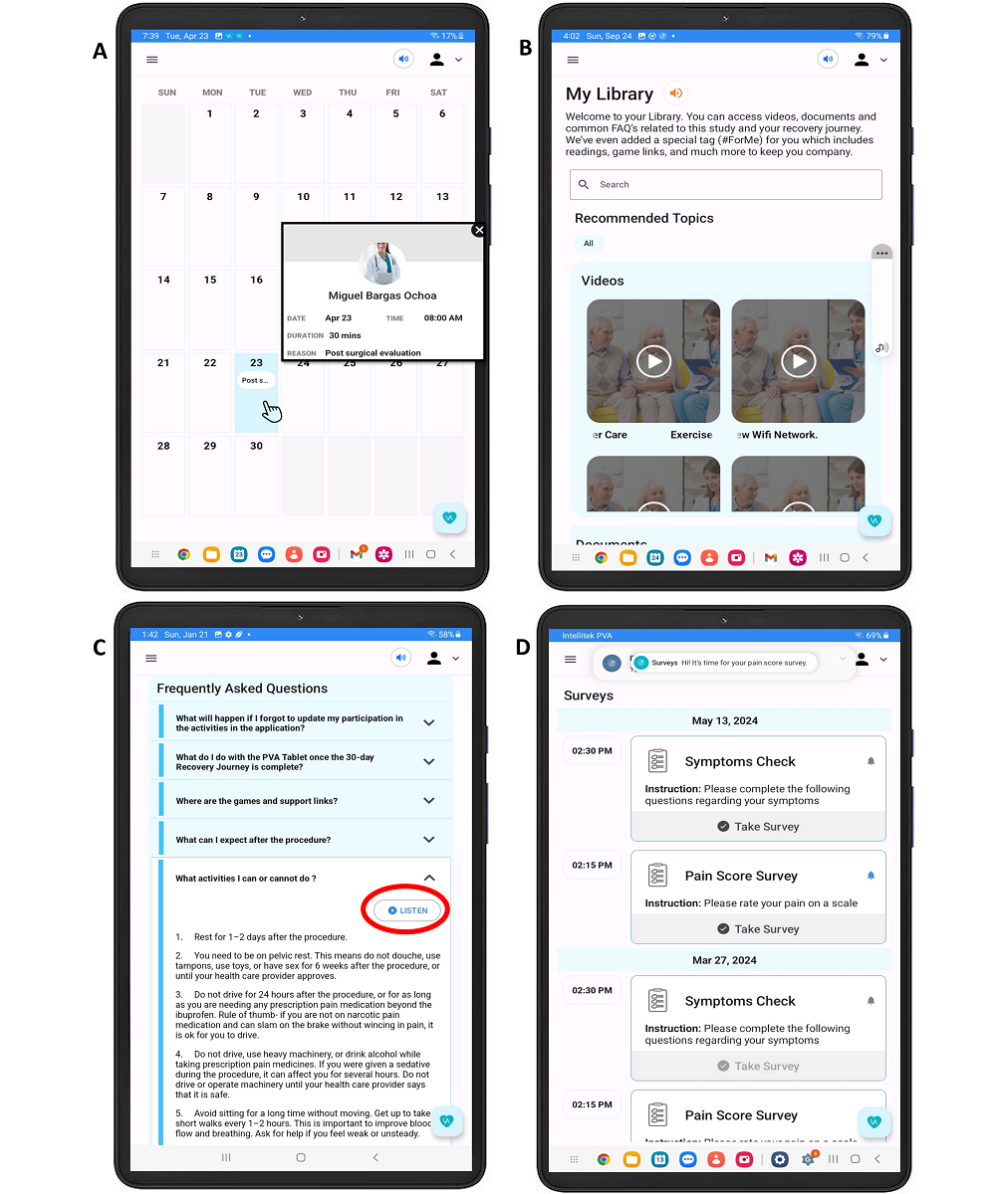
Personal Virtual Assistant displaying the patients’ portal additional navigation tabs. (**A**) Appointment tab displays a calendar. Details appear upon clicking on the date. (**B**) My Library contains educational content about Personal Virtual Assistant and surgical recovery. (**C**) Frequently Asked Questions about technology troubleshooting and recovery process includes audio option for listening to these explanations. (**D**) Surveys patient must complete that inquire about symptoms and pain.

On the top left side of the screen, a menu sidebar button is available to allow patients to navigate into other aspects of their care plan such as “Appointments,” “My Library,” “Messages,” and “Surveys,” (Figure 1B). By manually selecting or speaking aloud “Appointments” on the PVA, a calendar appears displaying upcoming clinic visits. Then, more details are available upon selecting the desired appointment (Figure 2A). The “My library” tab offers educational video content covering various topics such as guidance on how to use the tablet, exercise during cancer care, and guidance on postsurgical recovery (Figure 2B). Within this tab, a frequently asked questions section is also available, in which patients have the option to listen to them outload by pressing the “listen” button located above the text (Figure 2C). In addition, the “My Messages” tab opens a chat box where patients can communicate with the user behind the care provider portal, in this case the clinical research team. Finally, the “My Surveys” tab redirects to the home-screen symptom surveys.

**Figure 3.**
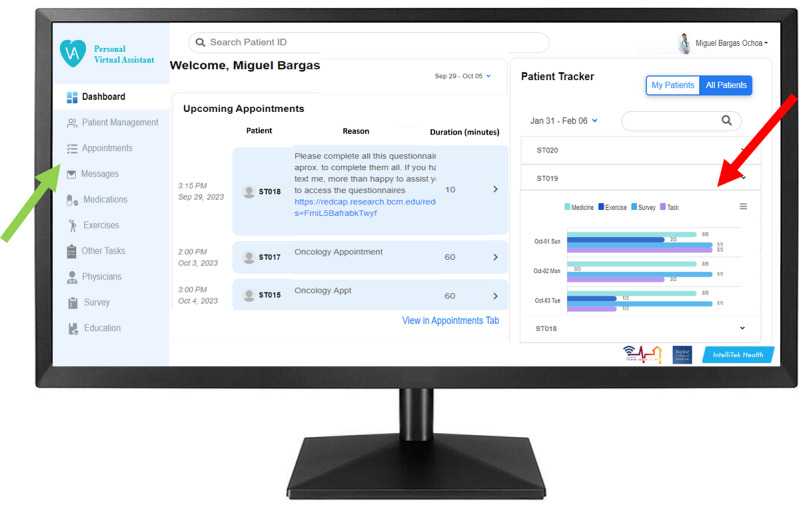
Personal Virtual Assistant displaying the care provider portal. Main dashboard on any computer with internet connection. Green arrow: points to the navigation sidebar. Red arrow: points at the adherence tracker, which opens a drop-down menu that shows daily adherence for each of the 4 categories in the care plan.

The Care Provider Portal is accessed through a website on any computer with an internet connection. In this study, the portal was personalized for use by the clinical research team. Once accessed by the user, a dashboard screen listing all patients with their upcoming appointments and weekly logins to the PVA system is displayed (Figure 3). On the left side of the screen, a menu sidebar including 10 different tabs allows the user to individually customize each patient care plan. For instance, in the “Patient Management” tab, the user can customize each patient’s posthospital discharge instructions. In the “Medications” and “Exercises” tabs, users can manage drug dose, frequency, duration, and mobility sessions, respectively. All information provided through these tabs are stored as repositories, feeding an electronic library for future care plans.

The cloud backend is a secure cloud-based platform that allows for instant updates to care plans, sends alerts, monitors adherence to care plans, arranges appointments, enables communication, and creates a resource library with relevant content for patients and care providers.

### Integration of Clinical Content Into the Personal Virtual Assistant

The PVA clinical content was developed by a multidisciplinary team including expert physicians in surgical oncology, medical oncology, pulmonology and critical care, and medical researchers from 1 academic institution (Baylor College of Medicine, Houston). As a result, specific discharge care content was created for 2 medical specialties: hepatobiliary surgery and gynecologic oncology. Engineers from 1 industry (Smartek21, Seattle) assigned the developed content into the different PVA features previously described.

**Figure 4.**
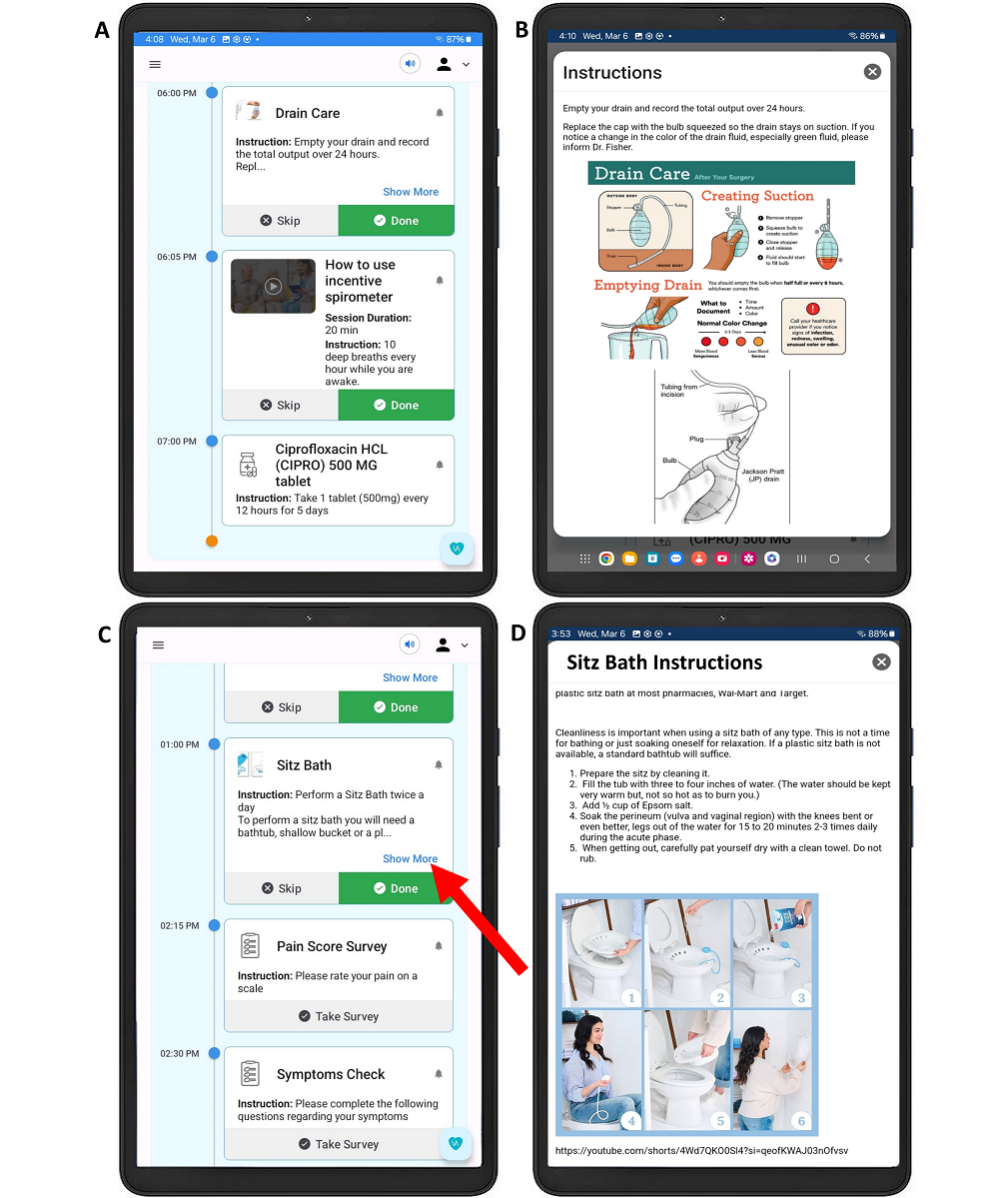
Personal Virtual Assistant displaying the patients’ portal “specific tasks” tab. (**A**) Home screen displaying the specific task “drain care” for patients undergoing hepatobiliary procedures. (**B**) Explanation of drain care (adapted from Go et al [17] which is published under a Creative Commons Attribution-NonCommercial International License [18]). (**C**) Home screen displaying the specific task “sitz bath” for patients undergoing gynecologic oncology procedures. (D) Upon clicking on the “show more button,” an explanation in the form of text and images appears. Image taken from SitzBliss sitz bath (SitzBliss in AMAZON).

The hepatobiliary surgery content was directed to pancreatic cancer procedures (ie, distal pancreatectomies and Whipple procedure). Content displayed in the home screen included prescribed medications, exercise guidance on respiratory therapy, symptom surveys, and specific tasks detailing wound care for surgical incisions, drain management, and video guides for self-administration of medications (Figure 4A and 4B).

The gynecologic oncology content focused on hysterectomies, radical vulvectomies, and cytoreductive surgeries. Content displayed in the home screen included prescribed medications, exercise guidance on lower extremity mobility (ie, walking, calf pumps, and leg raises), symptom surveys, and specific tasks detailing step-by-step incision care, and revision of instructions for healing of pelvic and genital areas (ie, sitz baths description, Figure 4C and 4D). In addition, information on postsurgery expectations, permissible and prohibited activities, warning signs requiring immediate medical attention, and recommended over-the-counter medications were included in the “My Library” tab.

In total, 2 symptom surveys were included in the content for both specialties, 1 monitored pain through a 10-point Likert scale, and the other monitored deep vein thrombosis (DVT) and assessed bleeding signs from mucosa or surgical incisions, using questions suggested by the clinical team.

### Study Design

To test the PVA’s feasibility, a 4-week pilot prospective study in individuals undergoing hepatobiliary and gynecologic oncology procedures was performed between November 2022 and October 2023.

Eligible patients were aged ≥18 years old; scheduled to undergo ambulatory surgery due to suspected malignancy; willing to engage with a PVA for 4 weeks following hospital discharge. Patients were excluded if they had: major foot and ankle problems (eg, major amputation and severe neuropathy); unable to provide informed consent; documented (confirmed through electronical medical records) major cognitive impairment, a psychiatric condition or abnormal laboratory results that, in the judgment of the clinical investigator, would interfere with the ability to participate in the study; active thrombotic condition or were using therapeutic anticoagulants; and were non-English speakers. In addition, individuals with uncorrected severe vision or hearing impairments that prevented them from effectively interacting with the PVA tablet, as determined by the investigators’ judgment, were excluded.

Before their surgery, patients received a 10-minute tutorial on PVA navigation. At hospital discharge (baseline), the research team manually customized the patients’ PVA content, incorporating the standard care plan detailed in their electronic medical record (EMR) by the attending surgeon. Demographic information was collected, and the patients were given the tablet with instructions to begin using it within 24hrs. After 4 weeks (end point), acceptability, and perceptions of the PVA were assessed by telemedicine, and adherence data was collected through a built-in tracking system within the PVA. Then, patients were asked to ship back the tablet. Weekly phone or video calls by the research team were performed during the study to address questions regarding the PVA functionality, content, or updates on their postoperative care according to their EMR.

### Feasibility, Adherence, and Acceptability Outcomes

Feasibility was assessed by quantification of dropout rate set to ≤10% throughout the study period (4 weeks). Adherence was quantified by completion of assignments (marked as “done”) in each of the 4 categories included in the care plan (ie, prescribed medications, exercises, symptom surveys, and specific tasks). For each patient, the percentage of each category was calculated by dividing the number of completed assignments by the number of days in which they were assigned during the study period.

Acceptability was assessed using a 12-item Technology Acceptance Model (TAM) questionnaire rated with a 5-point Likert Scale and ranging from strongly agree to strongly disagree, or very easy to very difficult. The TAM assessed 4 key areas: perceived ease of use, perceived usefulness, attitude toward use, and privacy concerns [19].

Furthermore, open-ended questions collected feedback on the user experience and suggestions for enhancing the PVA in Multimedia Appendix 1.

### Ethical Considerations

Patients signed an informed consent at the Dan L Duncan Comprehensive Cancer Center at Baylor College of Medicine before study enrollment. The protocol was approved by Baylor College of Medicine’s Institutional Review Board (protocol number: H-51654), and research procedures were conducted in accordance with the Declaration of Helsinki.

## Results

### Patient Characteristics

A total of 212 potential candidates were screened from the Duncan L Cancer Center at Baylor College of Medicine. Out of these, 166 were not eligible for surgery, 12 were non-English speakers, 7 did not respond to calls, and 7 declined to participate. This led to 20 eligible patients who consented and enrolled in the study, however 3 were withdrawn before study initiation due to surgery cancellation. In total, 17 patients initiated the study and received a tablet with the PVA app integrated. All patients were female (mean age 54.5, SD 12.7, years; mean BMI 33, SD 11.5, kg/m2). Out of these, 14 patients had gynecologic oncology procedures, and 3 had hepatobiliary oncology procedures. Ten out of 17 (59%) patients confirmed malignancy on biopsy (Table 1).

**Table 1.** Patient demographic information.

Characteristic			Participants (N=17)
Sex (female), n (%)			17 (100)
Age (years), mean (SD)			54.5 (12.7)
Race, n (%)			
	White		11 (6)
	Black		5 (29)
Ethnicity, n (%)			
	African American		5 (29)
	Caucasian		9 (52)
	Hispanic		2 (12)
	Asian		1 (7)
BMI, kg/m^2^, mean (SD)			33 (11.5)
Pathology report, n (%)			
	Benign (n=7)		
		Gynecological	7 (100%)
	Malignant (n=10)		
		Gastrointestinal	3 (30%)
		Gynecological	7 (70%)

### Feasibility and Adherence

One out of 17 patients (5.8%) dropped out from the study at postoperative day (POD) 3 due to health deterioration (reported feeling very weak and sick) and unwillingness to interact with the tablet. All other patients (16/17, 94.2%) completed the study and were included in the adherence and acceptability analyses. Among the analyzed patients, there was a mean adherence rate of 78% (SD 25%) for prescribed medications, 81 (SD 24%) for exercises, 61 (SD 30%) for symptom surveys, and 58 (SD 44%) for specific tasks. Detailed adherence data for each patient over the 4-week period is available in Multimedia Appendix 1. The responses to the open-ended questionnaire regarding technology acceptance, including perceived ease of use and perceived benefits, are available in Multimedia Appendix 2.

### Acceptability

There was an 80% patient endorsement across all TAM categories (Table 2 and Table 3). The highest endorsed items were regarding the simplicity of managing daily tasks (93.8%) and medications (93.8%), in the perceived ease of use category. Perception assessment is depicted in Multimedia Appendix 1.

**Table 2.** Responses (n=16) to perceived ease of use in the Technology Acceptance Model (TAM) questionnaire of patients who completed 4 weeks.

Perceived ease of use	Responses, n					Endorsement (very easy + easy), %
	Very easy	Easy	Neutral	Difficult	Very difficult	
Navigating my patient engagement app	11	1	3	1	0	75
Managing my appointments	11	2	1	2	0	81
Managing my medications	12	3	1	0	0	94
Managing daily tasks	11	4	0	1	0	94
Managing my messages	11	3	2	0	0	88
Connecting to my video calls	8	1	6	1	0	56
Accessing my exercise guidance	10	4	4	0	0	88

**Table 3.** Responses (n=16) to perceived usefulness, attitude toward use, and privacy concerns in Technology Acceptance Model (TAM) questionnaire of patients who completed 4 weeks.

Category and item		Responses, n					Endorsement (strongly agree + agree), %
		Strongly agree	Agree	Neutral	Disagree	Strongly disagree	
Perceived usefulness							
	I do not need the support of a technical person to use this app	10	1	1	3	1	69
	The patient engagement app is a useful resource in managing my post hospital care	8	4	4	0	0	75
	Reminders via the app effectively reminded me to complete my tasks	8	5	3	0	0	81
Attitude toward use							
	When this patient engagement app becomes available, I will use it	8	4	2	0	0	75
	I would recommend this patient engagement app to other friends or family members who are discharged from the hospital	8	5	3	0	0	81
Privacy concerns							
	I do not have privacy concerns while using this patient engagement app	10	0	4	1	1	62

### Reported Events During Study

Events were retrospectively collected via EMR in patients who completed the study. Three events were found; all were noted from patients undergoing gynecologic oncology procedures. One patient reported a falling incident to the research staff by phone call, but not through the PVA. This event did not require hospital readmission. Furthermore, 1 patient visited the emergency room (ER) on POD-17 due to sepsis secondary to acute cystitis or intra-abdominal abscess (per EMR note). Symptoms included pain during urination, lower back pain, and chills, which were not reported in the PVA chat. Symptom surveys recorded pain levels of 2/10 and 3/10 days before the ED visit. Treatment included computed tomography–guided needle aspiration, drain placement, and antibiotics. Another patient visited the ER on POD-28 due to lower abdominal pain and was diagnosed with an intramuscular rectus sheath hematoma (per EMR note). The patient experienced pain the previous week but did not report it in the PVA chat. Pain levels recorded in the PVA surveys were 7/10 just 2 days before the ER visit. The patient reported ineffective pain control through EMR chat (EPIC MyChart) and was advised to visit the ER by the health care team. Treatment consisted of analgesics and hospital observation for 24 hours.

## Discussion

### Principal Findings

This study explored the usability and acceptability of providing individualized postoperative care plans through a PVA system in patients undergoing hepatobiliary and gynecologic oncology procedures. This system is composed of 3 key elements, a tablet with an integrated app provided to the patient (patient portal), a website for care providers to manage from any computer (care provider portal), and a secure cloud backend system that links both portals. Through the care provider’s portal, the research team entered the patients’ postoperative care plans assigned by their attending physician which included guidance on daily personalized assignments divided into 4 categories (ie, medications, exercise, symptom surveys, and specific tasks). Then, a tracking system assessed the adherence to such categories. In total, 16 (93.7%) patients completed the study, showing an 80% acceptability rate evaluated through a technology acceptance model questionnaire. Adherence to the care plan surpassed 70% of assignments completed in the medications and exercise categories.

Digital health applications for postoperative recovery have shown efficacy for enhancing communication between patients and care providers in different areas [20,21]. Strategies used for such enhancement include symptom monitoring surveys [21-29] educational videos for wound care [17] and exercise implementation [30], which support patients in complying with their postoperative management. Other apps may alert care providers to detect warning signs for faster communication of adverse events [21,29,31]. Our multidisciplinary team integrated these features in an interactive, practical, and simple manner through a personalized tablet (Figure 2). This strategy ensures that compliance notifications remain separate from personal devices, thereby minimizing the risk of overlooking care management assignments. In addition, this system’s integrated “voice-enabled” navigation commands facilitate usability in those who have challenges on interpreting and inputting text. Emphasis was placed on presenting care plan assignments in an organized format, starting with the home screen, with the objective of creating a daily routine that patients could complete. Importantly, all features were integrated through direct consultation with clinicians, something we believe was crucial for patient engagement. Subhi et al [32] emphasized that without adequate professional input, digital health tools may deliver content that fails to meet patients’ needs and deploy interventions that are ineffective. Thus, the success of digital health tools relies on the proper combination of evidence-based systems with realistic content that involves individualized care plans guided by clinical minds.

Today, few authors have explored digital health applications for postsurgical oncologic care, all reporting high feasibility. Graetz et al [31] designed a mobile app for 26 patients undergoing gynecologic oncology procedures, incorporating progressive reminders regarding discharge instructions, medication adherence and completion of symptom surveys. The study was performed in 4 weeks, resulting in 88% of participants completing the study. In a similar population, Temple-Oberle et al [33] conducted a 6-week randomized controlled trial using a mobile app, in which patients uploaded wound pictures, drain volume data, reported symptoms or wound complications, and received unidirectional messages from their physicians. The approach was compared to standard of care, having only 1/36 patients (2%) in the intervention group dropping out of the study. Similarly, the majority of participants in the present 4-week study who used the PVA were those undergoing gynecological oncologic procedures, with 13/14 (93.7%) completing the study. We attribute this rate to the personalized content that was created for each patient. For instance, gynecologic patients undergoing surgery are recommended to perform sitz baths to relieve pain, swelling, and improve wound healing in the pelvic area [34]. However, proper instructions on the right equipment, water level and temperature, duration, and frequency of baths, are often forgotten [35]. The PVA reminds and guides patients to perform this task by notifications and banners on the screen (Figure 4C). This notification system has been shown to increase usability of digital health tools in gynecologic oncology patients [31]. Noteworthy, it is difficult to ascertain if the notification system influenced patient compliance and study completion the most. In addition, the known low-risk of postoperative complications in the group of gynecologic oncology patients could have been another factor contributing to a less pronounced dropout rate [31,33].

Another important aspect for assessing feasibility of digital health tools is the quantification of adherence to such systems. For instance, Mata et al [36] considered adherence as completion of 5 specific daily tasks (ie, early mobilization, gum chewing, consumption of oral liquids, breathing exercises, and consumption of protein drink) by following instructions from a tablet in 40 hospitalized patients who underwent colorectal surgery. Among these, 60% had a cancer diagnosis. Interestingly, a 94% adherence rate was seen on POD-1 but declined to 43% at POD-3. Similarly, Low et al [37] measured in-hospital adherence among patients undergoing abdominal cancer surgery, based on symptom survey responses via a mobile app and quantification of daily usage of a smart band (Fitbit). Adherence rates reported were 22% and 35%, respectively. However, when measuring adherence in the posthospital discharge phase, both rates increased (41 and 65%, respectively). This highlights how the timing and setting of digital health tools usage can impact adherence, particularly in those employed once patients have cleared hospitalization. In this study, posthospital discharge adherence was evaluated upon completing the assignments within the 4 care plan categories. This resulted in a high adherence rate for medications and exercises (78% and 81%, respectively). However, the patients’ freedom to mark each assignment as “done” could have biased our results, as it is difficult to verify whether these were truly completed. On the other hand, there was a 61% and 58% adherence rate for symptom surveys and specific tasks, respectively. Perhaps this was reflected on the 46% rate of patients who recommended including fewer surveys and questions in the PVA in Multimedia Appendix 1. Nonetheless, an interventional study evaluating clinical outcomes (ie, faster recovery, adverse events, and hospital readmission) is warranted to confirm associations with adherence.

To evaluate acceptability, the present study used a TAM questionnaire tailored for the PVA system, showing an 88.75% ease of use, 86.62% perceived usefulness, 85% attitude toward use, and 81% privacy concerns rates. No notable differences in technology acceptability were observed between younger (<60 years old) and older (>60 years old) patients, with overall scores appearing similar across age groups. While a small number in both groups indicated needing technical support or gave neutral responses regarding app usage, these differences were minimal and not statistically analyzed. Other cancer digital health apps using acceptability queries have shown similar high rates. Karlsson et al [38] utilized the System Usability Scale revealing a 77% ease of use score for an app that encourages mobility in patients recovering from abdominal cancer surgery (Pedatim, Phystec), surpassing the threshold (68 points) for high acceptability of this query [39]. Hwang et al [40] used the Patient Satisfaction Survey to evaluate an app that enables remote monitoring of wounds and communication with care providers in patients undergoing breast cancer surgery (Medeo), revealing a 90% ease of use, 90% attitude toward use, 95% perceived usefulness, and 100% of privacy concern rates. These results reflect the potential use of digital health tools in cancer patients recovering from surgical procedures and encourage researchers for future and continuous development of such systems.

Although the present study focused on exploring the PVA’s feasibility and acceptability, we sought to retrospectively collect incidents that happened during the study period to understand the challenges that can be addressed for future system improvement of the PVA. For example, one patient was readmitted to the hospital on POD-28 due to rectus sheath hematoma. Interestingly, this patient reported a poor adherence rate in all categories (39% for medications, 61% for exercises, 54% for symptom surveys, and 0% to specific tasks). Despite patients being instructed to directly communicate postoperative incidents or complications to their care providers, our system’s alternative monitoring options (ie, bidirectional chat and Likert pain scales) failed to collect such incidents. In fact, neither the patient’s care providers nor the PVA system had evidence of the three reported incidents. We attribute this to the lack of warning sign surveys evaluating fever, wound complications, urinary tract infections, hematomas, or ileus (in gynecologic procedures) [41]. We also believe that proper patient education on warning signs should be included in the home screen. In addition, the system could be equipped with advanced monitoring features such as vital signs (ie, temperature, heart rate, and respiratory rate) and symptom reporting surveys integrated with automated alarms to alert users when certain thresholds are exceeded. These technological enhancements could facilitate prompt detection of postoperative complications to avoid hospital readmissions.

The study findings highlight opportunities to enhance the clinical application of this interactive digital technology, particularly in improving adherence to symptom reporting and supporting the successful completion of specific tasks, that may benefit from more detailed, step-by-step education incorporating comprehensive images or videos. Acceptability and usability are essential first steps in deploying interactive digital solutions to support posthospital discharge care plans. While these solutions hold promise for preventing surgical complications and reducing hospital readmissions, such outcomes remain the ultimate goals of these technologies and require further validation. Research suggests that remote patient monitoring during postsurgical recovery phases can significantly lower hospital readmissions and minimize unnecessary clinic visits compared to standard of care [40]. We speculate that the PVA could meaningfully improve clinical outcomes by enhancing patient adherence to postdischarge care plans, including medication adherence, exercise routines, symptom reporting, and task completion. However, this speculation requires validation through future interventional studies. Further research should also explore how the PVA can optimize communication between patients and care providers, fostering a more seamless and effective recovery process.

### Limitations

There are several limitations to our study. Major limitations of this study are its exploratory design, small sample size, and the absence of a control group. These factors restrict our ability to evaluate the PVA’s effectiveness compared to standard postdischarge care, limiting our understanding of its overall impact. The low dropout rate and high adherence observed could be attributed to the weekly monetary compensation offered to patients, which may have influenced their participation levels. The questions included in the TAM for evaluating participant’s adherence and perceptions might have introduced an acquiescence bias, with patients predisposed to agree with the assessment statements. In addition, we did not examine variations in technology acceptance across different age groups, ethnicities, or cancer types. Such an analysis could provide valuable insights and broaden the applicability of our findings. In the next phase of the study, we plan to incorporate demographic analysis to better understand PVA acceptability and tailor its use to diverse patient populations. Another significant limitation was the lack of integration between the PVA system and electronic medical records, requiring research teams to manually enter each participant’s discharge plan. This manual process was not only time-consuming but also involved transcribing detailed instructions in each category of the care plan. Even though the system adherence tool identifies which specific assignments within each category of the care plan (ie, medications, exercise, tasks, and surveys) have been completed by the patient, the clinical study coordinator or health care provider must continuously revise this data on a daily basis. In addition, the health care provider portal, although intended for provider use, was managed by the research team. Thus, the absence of direct contact between patients and health care providers could have affected their experience or willingness to engage fully with the PVAs messaging tool. Regarding the surveys used in the PVA (ie, pain scales and DVT symptoms), patients’ answers require continuous revision on a daily basis by the user. However, the research team only reviewed these surveys at the end of the study, highlighting a critical limitation. The absence of real-time symptom collection likely affected the ability to assess the PVA’s effectiveness in early prevention and detection of common postsurgical complications.

To address this limitation, future iterations will focus on redesigning surveys to facilitate real-time symptom reporting and efficient review by the clinical care team. This redesign aims to enhance usability while avoiding time-consuming processes, ultimately improving the PVA’s role in patient care and early intervention. Furthermore, the app requires internet connection to function and receive real-time updates to the care plan. At the time of this study, it was not available on Google Play Store or Apple platforms, which meant that updates had to be manually downloaded through a link provided by our industry partner (Smartek21) from the web. Finally, while the PVA allows for picture uploads through a secure chat with researchers, this feature was not instructed as part of the study protocol. We believe this feature should be used in future studies, as it is crucial for the prompt identification of clinical warning signs [21,22,24,25,27-29,42].

### Conclusion

This exploratory study demonstrated the usability and acceptability of an interactive digital solution designed to provide an organized, step-by-step guide for postsurgical care, and simplify adherence to care plans in patients undergoing gynecological oncology and hepatobiliary oncology procedures. The findings suggest perceived acceptability, ease of use, and intention to use the platform, although privacy concerns remain a limitation for broader scalability. The results showed fair-to-good adherence to certain postdischarge tasks, such as recommended exercises and prescribed medications, while adherence to symptom surveys and specific tasks was notably lower. Integrating additional features, such as notification reminders and voice-enabled systems, appeared promising for improving compliance. Furthermore, information retrospectively gathered from postsurgical complications provided valuable insights for enhancing the PVA system in future iterations. These findings underscore the potential for interactive digital health solutions to improve communication between patients and care providers while coaching patients to adhere to prescribed postdischarge tasks, which may ultimately enhance recovery outcomes. However, these observations are preliminary and need to be confirmed in studies with larger sample sizes. In addition, future research should focus on validating the effectiveness of this solution in improving posthospital discharge outcomes.

## Supplementary material

10.2196/64145 Multimedia Appendix 1 Feedback from open-ended questions on the user experience and suggestions for enhancing the Personal Virtual Assistant.

10.2196/64145 Multimedia Appendix 2 Responses to the open-ended questionnaire regarding technology acceptance, including perceived ease of use and perceived benefits.

## Abbreviations

DVT: deep vein thrombosis
EMR: electronic medical record
ER: emergency room
POD: postoperative day
PVA: Personal Virtual Assistant
TAM: Technology Acceptance Model
