# Supplementary material for: Development and Implementation of a Personal Virtual Assistant for Patient Engagement and Communication in Postsurgical Cancer Care: Feasibility Cohort Study
Source: JMIR Cancer. 2025 Feb 18;11:e64145. doi: 10.2196/64145 (PMC11855163; doi:10.2196/64145)
Supplement: Multimedia Appendix 1 [file cancer-v11-e64145-s001.docx]

|  |  |
| --- | --- |
| **Question** | **Answer/outcome** |
| **Recommended changes to the PVA** | Seven out of fifteen (46%) patients recommended changes |
|  | - Lighter tablet - Fewer surveys and questions - Better education on usage - Option to change responses and change entry errors. - Using apple rather than android - Add the ability schedule the timing of medication themselves. |
| **Health Changes** | Nine out of fifteen (60%) responders felt their health changed during the third week. |
|  | - Eight reported self-improvements, experiencing healing and increased strength. - One patient reported experiencing more pain |
| **Physical activity improvements** | Eleven out of fifteen (73%) responders agreed their level of physical activity improved in the last week compared to the third week |
|  | - 2/15 (13%) responded “somewhat or slightly agreed” |
| **Overall Experience** | Eight out of fifteen (53%) reported positive feedback |
|  | - Described the app as “very helpful,” “worked fine and had no issues,” “enjoyed using the app,” “would highly recommend it", and “liked the encouraging words and ease of use.” |
|  | Some provided suggestion for improvement such as:   - Use plainer language that ordinary people use. - Make the questions clear and more specific. - Allow a time stamp on task completion and ability to add comments - One patient noted that there was a discrepancy between the doctor's discharge instructions and the hospital's generic discharge instructions. |
