# Supplementary material for: Development and Implementation of a Personal Virtual Assistant for Patient Engagement and Communication in Postsurgical Cancer Care: Feasibility Cohort Study
Source: JMIR Cancer. 2025 Feb 18;11:e64145. doi: 10.2196/64145 (PMC11855163; doi:10.2196/64145)
Supplement: Multimedia Appendix 2 [file cancer-v11-e64145-s002.docx]

|  | | | | |
| --- | --- | --- | --- | --- |
| **ID** | **Medications** | **Exercises** | **Surveys** | **Tasks** |
| ST001 | 20/28 (71%) | 20/28 (71%) | 0/7 (0%) | 0/0 |
| ST002 | 13/27 (48%) | 5/5 (100%) | 2/24 (8%) | 0/0 |
| ST003 | 27/27 (100%) | 20/23 (87%) | 12/15 (80%) | 1/1 (100%) |
| ST004 | surgery cancelled |  |  |  |
| ST005 | surgery cancelled |  |  |  |
| ST006 | 3/28 (11%) | 1/25 (4%) | 4/7 (57%) | 1/28 (4%) |
| ST007 | surgery cancelled |  |  |  |
| ST008 | 25/28 (89%) | 25/27 (93%) | 24/28 (86%) | 25/28 (89%) |
| ST009 | 18/27 (67%) | 17/26 (65%) | 7/27 (26%) | 16/27 (59%) |
| ST010 | 26/28 (93%) | 26/28 (93%) | 15/25 (60%) | 0/28 (0%) |
| ST011 | 27/27 (100%) | 27/27 (100%) | 0/0 | 27/27 (100%) |
| ST012 | 24/26 (26%) | 25/26 (96%) | 22/2 (85%) | 23/26 (88%) |
| ST013 | 19/25 (76%) | 19/25 (76%) | 7/20 (35% | 21/28 (75%) |
| ST014 | 3/3 (100%) | 27/27 (100%) | 27 (100%) | 0/27 (0%) |
| ST015 | 27/27 (100%) | 24/24 (100%) | 21 (84%) | 26/26 (100%) |
| ST016 | 9/10 (90%) | 27/28 (100%) | 20/28 (71%) | 27/27 (100%) |
| ST017 | 11/28 (39%) | 17/28 (61%) | 14/26 (54%) | 0/27 (0%) |
| ST018 | 19/23 (83%) | 17/22 (77%) | 16/22 (73%) | 0/22 (0%) |
| ST019 | 26/27 (96%) | 21/27 (78%) | 26/27 (96%) | 26/27 (96%) |
| ST020 | Dropout |  |  |  |
| **Total Average (SD)** | 78% (25%) | 81% (24%) | 61% (30%) | 58% (44%) |
